# Did aging-related beliefs and behaviors change during the COVID-19 pandemic?

**DOI:** 10.1007/s10433-026-00929-6

**Published:** 2026-07-07

**Authors:** Sonja Radoš, Maria K. Pavlova, Klaus Rothermund, Rainer K. Silbereisen

**Affiliations:** 1 Institute of Gerontology, University of Vechta, Vechta, Germany; 2 Department of Psychology, Friedrich Schiller University of Jena, Jena, Germany

**Keywords:** COVID-19, Political attitudes, Socioeconomic resources, Successful aging, Views on aging

## Abstract

The COVID-19 pandemic reinforced the narrative of old-age vulnerability and disengagement. Using data from a nationally representative sample of German adults (*N*_T1_ = 2,007, *N*_T2_ = 733; age range 16–94 years), we investigated changes in aging-related beliefs and behaviors between 2016 and 2022. Specifically, we considered perceived expectations for active aging (PEAA), positive age stereotypes, and preparation for age-related changes in the domains of physical health, mental health, and social engagement. The average PEAA and positive age stereotypes significantly decreased across age groups and domains whereas preparation for age-related changes decreased slightly and only in the social engagement domain. Residual change analyses revealed a greater reduction in PEAA across domains among respondents with (vs. without) children in the household. In those with more pronounced populist political beliefs, PEAA in the social engagement domain diminished more. None of the focal predictors predicted change in positive age stereotypes. Surprisingly, working in an occupation disrupted by the pandemic (compared with relatively undisrupted occupations) predicted more positive change in preparation for age-related changes in the mental health domain. Overall, the public discourse regarding older people and old age during the pandemic may have contributed to the worsening of aging-related beliefs. The stability in preparation suggests that such behaviors served to adapt to the unique circumstances of the pandemic.

**Supplementary Information:**

The online version contains supplementary material available at 10.1007/s10433-026-00929-6.

Active aging policies encourage older adults to maintain healthy behaviors and participate in social, economic, and civic affairs (World Health Organization 2002). However, the COVID-19 pandemic led to political decisions that restricted the opportunities for active lifestyles (European Parliament 2021), while older adults were widely portrayed as vulnerable (Swift and Chasteen 2021). Arguably, these developments revived the already discarded narrative of disengagement in older age (Cumming and Henry 1961), and ageism could increase accordingly (Sutter et al. 2022; Werner and AboJabel 2023). Indeed, research has indicated a change toward less favorable aging-related beliefs and feelings during the pandemic (Anderson and Gettings 2022; Seifert 2021; Wahl et al. 2022). In the present study, we investigated the mean changes in multiple aging-related beliefs and behaviors—perceived expectations for active aging (PEAA), positive age stereotypes, and preparation for age-related changes—between 2016 and 2022 in Germany. Additionally, we considered the potential predictors of individual differences in change, namely indicators of socioeconomic disadvantage and political attitudes.

Age stereotypes are socially shared beliefs about older people and aging. They can be descriptive (i.e., what older people are like; Levy 2009) or prescriptive (i.e., how they should behave; North and Fiske 2013). Both types may relate to oneself and others, and both vary systematically across life domains (Kornadt and Rothermund 2011; Rothermund and de Paula Couto 2024). Unlike disengagement prescriptions (Cumming and Henry 1961), PEAA reflect self-related prescriptive age stereotypes of activation (e.g., “It is expected of me to be physically fit”; Pavlova and Silbereisen 2012). Both descriptive and prescriptive age stereotypes can shape how individuals treat older people and approach their own aging (Rothermund and de Paula Couto 2024). Furthermore, preparation for age-related changes (hereafter simply “preparation”)—i.e., behaviors that address anticipated aging-related scenarios in different life domains (Kornadt and Rothermund 2014)—has been linked to health and leisure activities in older age (de Paula Couto et al. 2022). Together, these aging-related constructs can reflect in how far the notion of active aging resonates with the public.

Driven by demographic change and a growing recognition of older adults’ contributions, active aging was adopted as a policy goal across European countries in the last two decades, though to varying degrees (European Parliament 2021; World Health Organization 2002). In Germany, governmental, community, and research initiatives were sustained since the early 2000s; accordingly, the Active Aging Index steadily rose until 2018 (Tesch-Römer and Wurm 2012; United Nations 2019). Furthermore, between 1998 and 2014, descriptive age stereotypes in Germany were found to become more gain- and less loss-oriented (Beyer et al. 2017), whereas awareness of expectations for active aging was reportedly present both in 2012 and in 2016 (Pavlova and Silbereisen 2012; Pavlova et al. 2023). Despite the age-graded change in self-perceptions of aging in late adulthood (Diehl et al. 2021) and regional trends toward more negative self-perceptions of aging in faster-aging German districts (Wolff et al. 2018), active aging appeared salient and aging-related beliefs seemed increasingly positive in Germany prior to the pandemic.

With the onset of the COVID-19 pandemic, active aging campaigns were sidelined across Europe (European Parliament 2021). Many national pandemic-control policies, including in Germany, prioritized protecting older adults’ health at the cost of social integration due to their heightened risk of severe COVID-19 outcomes (Molani et al. 2022). Age-segregated policies—alongside media commentaries—showcased older age as a phase of vulnerability and older adults as requiring protection (Myrczik et al. 2023; Swift and Chasteen 2021). Arguably, these developments also reintroduced disengagement norms, according to which older adults should withdraw from social roles, interactions, and commitments (Cumming and Henry 1961). Indeed, social isolation and loneliness among older adults increased during this time (Su et al. 2023). Overall, pandemic conditions appeared not only to undermine earlier efforts to associate older age with active lifestyles but also to reinforce the negative images of older adults and aging more broadly (cf. Anderson and Gettings 2022).

In Germany, there were three nationwide lockdowns in 2020 and 2021 lasting two to six months each. With some regional variation, schools, daycare centers, and non-essential businesses closed, and public gatherings were prohibited. Healthcare facilities implemented strict infection control, and vaccination was prioritized for older people and healthcare workers. Sometimes, a negative COVID-19 test or vaccination certificate was required to access public venues. The government consistently emphasized interpersonal and intergenerational solidarity, and these appeals were largely followed by the public. Solidarity practices, however, decreased during the second lockdown (Schönweitz et al. 2024). Older adults also faced calls to take responsibility by self-isolating (Ellerich-Groppe et al. 2021). Restrictive measures gradually eased throughout 2022 and ended in early 2023.

Early in the pandemic, both personal and general views on aging—particularly in the health domain—became increasingly negative among German older adults (Wahl et al. 2022; Wettstein et al. 2023a), a trend observed in other countries as well (e.g., Seifert 2021). At the same time, most German adults in the second half of life did not report elevated levels of perceived age discrimination (Wettstein and Nowossadeck 2023). Regarding the behavioral indicators of active aging, physical activity declined nationwide in 2020, while sedentary activities increased (Herbolsheimer et al. 2024). Interestingly, however, the amount of time middle-aged and young-old adults spent volunteering remained stable between 2017 and 2021 (Simonson and Kelle 2023). Nevertheless, mental health declined in the German population during the entire pandemic period (Patzina et al. 2025).

These findings point to the importance of examining different aspects of aging-related beliefs and behaviors. A domain-specific approach captures gains and losses in different life domains (e.g., leisure, family, health), thereby providing a differentiated understanding of the aging process (Kornadt and Rothermund 2011) and enabling the prediction of specific later-life outcomes (Kornadt et al. 2020). We considered the domains of physical health, mental health, and social engagement for several reasons. First, the active aging policy framework emphasizes health maintenance and participation in later life (World Health Organization 2002). Second, these domains were among the most directly affected by policy measures and health threats during the pandemic. Finally, previous research supported the differentiation between age stereotypes, preparation, and PEAA across these domains (i.e., different mean levels, Kornadt and Rothermund 2011; Kornadt and Rothermund 2014; Pavlova et al. 2023; different predictors, Kornadt and Rothermund 2014; Kornadt et al. 2020; Pavlova et al. 2023; Radoš et al. 2025). Drawing on the prior pandemic-related research and our knowledge of the German context (e.g., Herbolsheimer et al. 2024; Sutter et al. 2022; Wahl et al. 2022; Werner and AboJabel 2023), we expected a negative mean change in aging-related beliefs and behaviors across the domains of physical health, mental health, and social engagement in 2022 compared with 2016 (Hypothesis 1).

Guided by the well-established notion that (abrupt) social change affects people unequally (Elder 1974; Pinquart and Silbereisen 2004), we also investigated individual differences in the change in aging-related beliefs and behaviors. Overall, we assumed that people who suffered more under the pandemic circumstances might have resented older adults—the protection of whom was the declared aim of most policy measures—or developed a more pessimistic outlook on aging in general. Socioeconomic disadvantage—whether preexisting or induced by the pandemic—may foster more negative aging-related beliefs and behaviors (Werner and AboJabel 2023). In Germany, financial strain particularly affected working middle-aged and older adults with low pre-pandemic income (Romeu Gordo et al. 2023). On the one hand, in the context of (intergenerational) solidarity during crises, economic hardship may even foster communal orientation (Kraus et al. 2012). On the other hand, in a large-scale international study, economic threats during COVID-19 were associated with decreased preventive health behaviors through lower prioritization of communal values (Lemay et al. 2023). Moreover, socioeconomic disadvantage—including low education—was associated with ageism in the USA (Allen et al. 2022), although *subjective* pandemic-induced economic threat did not predict negative age stereotypes in Canada and the USA (Sutter et al. 2022). Furthermore, working conditions were differentially affected during COVID-19: Whereas some Germans experienced reductions in working hours, others—particularly those in the public sector and essential workers—experienced the opposite (Engstler et al. 2023). For instance, healthcare workers were understaffed, overworked, and immediately exposed to the health consequences of COVID-19 for older adults. Lastly, people in charge of children experienced increased distress as school and daycare closures imposed additional childcare responsibilities on them (Li et al. 2022).

In sum, we expected low educational attainment (Hypothesis 2), having children in the household (Hypothesis 3), and income reduction (Hypothesis 4) to predict negative change in aging-related beliefs and behaviors across domains. Similarly, we expected working in healthcare to predict negative change, particularly in the physical health domain (Hypothesis 5a). More generally, we anticipated that working in occupations that were disrupted by the pandemic (see Supplement 1 for the categorization of occupations) would predict negative change in aging-related beliefs and behaviors across domains (Hypothesis 5b).

Throughout the pandemic, the political climate in Germany was highly polarized. The government consisted of centrist parties that formulated policies based on the known age differences in health risks. This prioritization of older adults may have exacerbated intergenerational tensions (cf. North and Fiske 2013). In particular, those who disagreed with the pandemic-control policy may have resented older people or held them responsible for the imposed restrictions. This resentment could exist alongside a general positive regard for older adults. For example, right-wing political leaning was associated with higher warmth perceptions of older adults among American and Canadian adults, yet frustration with social distancing measures predicted seeing older people as a burden on collective resources (Sutter et al. 2022). To gauge the disagreement with the policy, we considered support for non-centrist (i.e., oppositional) parties and affinity for populism since dissatisfaction with pandemic-control policies has been linked to populist beliefs (Ehrke et al. 2023). We therefore hypothesized that a stronger affinity for populism (Hypothesis 6) and stronger support for non-centrist political parties (Hypothesis 7) would predict more negative change in aging-related beliefs and behaviors across domains.

We conducted our analyses on an adult sample spanning early adulthood to advanced old age (16–94 years of age). Understanding change in aging-related beliefs and behaviors from a life-span perspective is important, because they continuously shape developmental trajectories and outcomes (Kornadt et al. 2020; Popham et al. 2011). Before the onset of older age, old-age stereotypes inform subjective expectations about later life and can act as self-fulfilling prophecies (Levy 2009). This is concerning given the increase in negative feelings about aging reported by young adults after the pandemic (Anderson and Gettings 2022). Moreover, previous research found moderately high levels of PEAA in adults of all ages (Pavlova et al. 2023). In addition, while the level of preparation increases with age, adults aged 30–49 years were shown to engage in preparatory activities for changes they anticipate will occur in later life (Kornadt and Rothermund 2014). Still, as the consequences of the pandemic varied across age groups, we explored age differences in the mean change and repeated our regression analyses on a subsample of adults aged 55 and over. Moreover, health should be generally more salient to older adults because of the age-related health decline, but at the same time, older adults with better health felt less threatened by the pandemic and were more resistant to vulnerability narratives (Wettstein et al. 2023b). We therefore explored the interaction between general health and age without directional hypotheses.

## Method

### Participants and procedure

We used data from the SOEP-IS, the multi-disciplinary representative annual survey of German adults (age 16 and older), which is intended for innovative user-designed research projects (SOEP 2024b). Participants provide informed consent and receive monetary incentives; data collection complies with ethical research standards (SOEP 2024a). Before the pandemic, trained interviewers conducted standardized computer-assisted personal interviews. Since 2020, interviews have mostly been conducted by phone (SOEP 2024b). According to the fieldwork reports (SOEP 2024b), 3049 households participated in 2016 (85.9% of the gross sample) while 1803 households completed the interviews in 2022 (97.3% of the gross sample). Our items on PEAA, age stereotypes, and preparation were administered to the randomly drawn subsample of 2007 individuals (aged 16–94) in 2016. In 2022, our items were administered for the second time to a portion of the original subsample and to an additional 80 participants (total *N* = 733). Sample-size reduction between 2016 and 2022 was largely driven by dropout and only to a small extent by mortality. Compared to T_2_ participants, those who dropped out of the longitudinal sample had lower objective and subjective socioeconomic status, lower preparation levels across domains, and less positive age stereotypes in the mental health and social engagement domains at T_1_. We used predictors from different waves between 2016 and 2022 for our sample based on data availability and/or wave-specific relevance (e.g., pandemic-related income change). Table 1 and Supplement 2 present the descriptive statistics, and Supplements 3–4 present correlations between predictors and outcomes.

**Table 1:** Descriptive statistics: predictors at T_1_

Predictor	Summary statistics	
	All T_1_ participants (*N* = 2007) at T_1_	Only T_2_ participants (*N* = 733) at T_1_
*Control variables*		
Age (16–94)^a^, *M* (*SD*)	52.3 (18.2)	49.8 (17.8)
Female, *n* (%)	1039 (51.8%)	368 (50.8%)
Subjective socioeconomic status (1–10), *M* (*SD*)	6.0 (1.5)	6.2 (1.3)
Cohabiting with a partner, *n* (%)	1313 (65.4%)	452 (62.3%)
General health (1–5), *M* (*SD*)	3.4 (0.9)	3.4 (0.8)
*Socioeconomic predictors*		
Educational attainment in years (7–18), *M* (*SD*)	12.2 (2.6)	12.9 (2.8)
*Occupation, n (%)*		
Less disrupted by the pandemic	86 (14.5%)	50 (14.2%)
Teleworking	168 (28.4%)	105 (29.8%)
Most disrupted by the pandemic	170 (28.7%)	102 (29.0%)
Healthcare	66 (11.1%)	38 (10.8%)
Manufacturing	102 (17.2%)	57 (16.2%)
Income change (-4500–4000 EUR), *M* (*SD*)	91.6 (568.1)	101.5 (555.9)
Children at home between 2020 and 2022, *n* (%)	266 (26.5%)	149 (20.6%)
*Political attitudes*		
Affinity for populism (1–5), *M* (*SD*)	3.4 (0.7)	3.3 (0.8)
Centrist-party preference, *n* (%)	347 (59.5%)	192 (56.1%)
No party preference, *n* (%)	555 (48.8%)	300 (46.7%)
Strength of support (0–5), *M* (*SD*)	1.8 (1.8)	1.9 (1.8)

Some items were administered to an independently selected random subsample in the waves between 2016 and 2022 or represent a combination of data from different waves. Individual missings did not exceed 5% in either wave. Due to inapplicable responses coded as missing values, occupation had 28% missing values at T_1_ and 40.7% at T_2_

^a^Age was calculated based on the 2016 data. Hence, the range includes participants who only took part in the 2022 survey wave but were underage in 2016

### Measures

#### Outcome variables

In each domain, three single items assessed PEAA (e.g., social engagement: “People have high expectations that I get involved in social and non-profit-making activities”), positive age stereotypes (e.g., physical health: “Older people are healthy and physically fit”), and preparation for age-related changes (e.g., mental health: “I actively take care to keep my mental fitness in older age, e.g. by mental activity in solving crossword puzzles or reading books and magazines”) with a 7-point rating scale (1 = *does not apply at all*, 7 = *applies completely*). Note that not all items included an explicit reference to aging, but the topic of aging was introduced at the beginning of the module (see Supplement 5 for the measurements in German with English translation). The items on PEAA were adapted from the Jena Study on Social Change and Human Development (Pavlova and Silbereisen 2012; Silbereisen et al. 2010), and the items on age stereotypes and preparation were adapted from the original multidimensional questionnaires by Kornadt and Rothermund (2011, 2014). In both waves, intercorrelations of the three items referring to the same construct were more closely related to each other than to the items from the other constructs, confirming their convergent and discriminant validity (Pavlova et al. 2023; see also Supplements 3–4). Discriminant validity of the items belonging to the same construct (e.g., PEAA) was further supported by domain specificity of some of their predictors (Pavlova et al. 2023; Radoš et al. 2025).

#### Socioeconomic predictors

To assess educational attainment, we used a generated variable measured in years of education or training. We employed 2020 data and replaced missing values with the available 2016 data. For occupations, we used a generated variable from 2020 based on the NACE industry-sector classification (Hartmann and Schütz 2002). Drawing on the findings regarding the impact of the pandemic on various work situations in Germany (Engstler et al. 2023), we categorized occupations by sector and the type of disruption during the pandemic (i.e., 0 = *less disrupted by the pandemic*, 1 = *teleworking*, 2 = *most disrupted by the pandemic*, 3 = *healthcare*, 4 = *manufacturing*; see Supplement 1). In regression analyses, occupational categories were dummy-coded, with the “less disrupted” serving as the reference category. To assess changes in income, we calculated the raw difference in household income per person between 2020 and 2022. Positive scores indicated higher household income in 2022 compared with 2020. Lastly, having children (aged 16 and below) in the household was a binary variable (0 = *no*, 1 = *yes*) based on the 2020 and 2022 data.

#### Political attitudes

To assess affinity for populism, we used a mean score of 12 items by Albers et al. (2023) administered in 2019 (e.g., “Ordinary people all pull together”, “People like me have no influence on what the government does”, 1 = *does not apply at all*, 5 = *fully applies*; *α* = 0.87). We assessed political party preference in 2020 with the questions “Do you lean toward a particular party?” and “Which party do you lean toward?” (0 = *centrist (CDU/CSU/SPD*), 1 = *all others*). Additionally, the participants rated the strength of their support for the preferred political party (0 = *no support*, 5 = *very strong support*).

#### Control variables

We controlled for chronological age, sex, and the correlates of PEAA known from previous studies on the same dataset: partnership, subjective socioeconomic status, and general health (Pavlova et al. 2023).

### Analytical approach

We used two complementary approaches to test our hypotheses regarding changes in aging-related beliefs and behaviors: Mean-level change and residual change analyses. Specifically, to test Hypothesis 1, we compared the mean levels of domain-specific PEAA, age stereotypes, and preparation between 2016 and 2022 using ANOVA in SPSS and the Wald test in Mplus 8.5 (Muthén and Muthén 2017). First, we conducted a repeated measures ANOVA in SPSS (28.0) with time and domain as within-subject factors and age group as a between-subject factor. This analysis showed the magnitude of mean-level change and estimated whether it differed across domains or age groups. Because of the multiple significance testing, we used an adjusted significance threshold (*p* < 0.01). Second, essentially the same analysis in Mplus served to check whether the estimates of mean-level change would hold if missing values were accounted for with a full-information estimator.

To test Hypotheses 2–7, we conducted residual change analyses in Mplus 8.5 using a maximum likelihood estimation with robust standard errors. Residual change analysis isolates the effects of predictors on the residual variance (i.e., variance not explained by the outcome value at T_1_) and therefore enables the conclusion that Variable X predicts the change in Variable Y in a particular direction. Controlling for the respective 2016 scores, we regressed each of the outcomes from 2022 on the predictors entered in blocks (i.e., only the control variables, the controls and socioeconomic predictors, the controls and political attitudes, and all of the predictors) for each domain separately. For significant effects in at least one domain, we compared the standardized regression coefficients between domains using a *z*-test. For Hypothesis 7, we computed the interaction between political party preference and the strength of party support. The main effect of party support showed how the strength of the support for centrist parties was related to residual change in the outcomes whereas the interaction showed whether this effect differed for the supporters of non-centrist parties. For the interaction between health (grand-mean centered) and age in the explorative analyses, we dichotomized the age variable (under 55 and 55 +), drawing on the approximate age threshold for the increase in health-related risks of COVID-19 (Molani et al. 2022). We used the full information maximum likelihood estimation to handle missing values (Muthén and Muthén 2017).

In supplementary analyses, to assess the generalizability of our findings to the German population, we repeated both the mean-level change analyses and the final regression model with weighted data. We also explored age differences (under 55 vs. 55 +) in the final regression model and conducted a sensitivity analysis to probe whether the associations between the predictors and the outcomes at T_1_ differed between participants who remained in the survey and those who dropped out. Here, we used an omnibus Wald test to estimate whether the regression model differed significantly across groups. For all supplementary analyses excepting weighted analyses, we used an adjusted significance threshold for post-hoc testing (*p* < 0.01).

## Results

### Mean comparisons and age differences

**Fig. 1:**
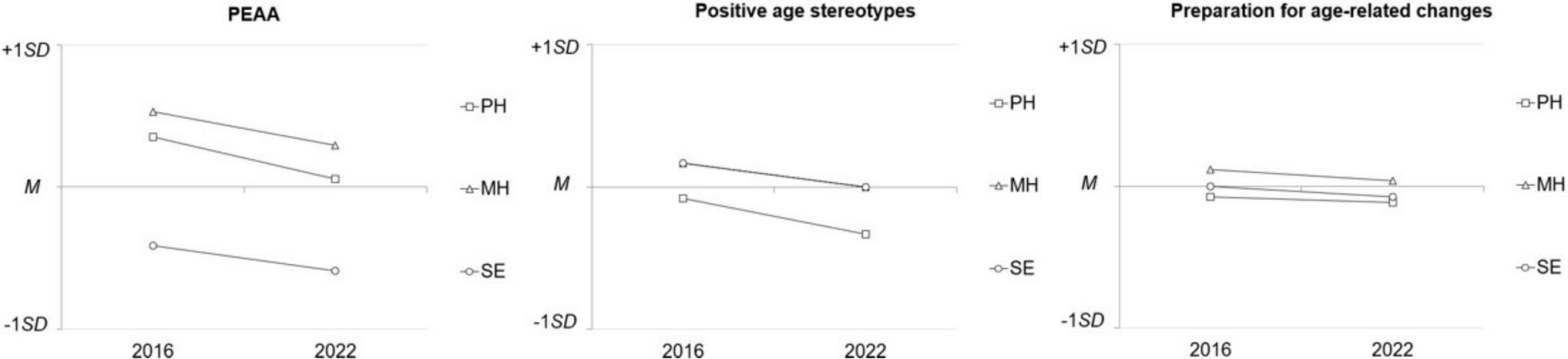
Mean comparisons by domain in PEAA, age stereotypes, and preparation for age-related changes between 2016 and 2022. *Note*. The y-axis was scaled to two pooled baseline *SD*s of the outcomes, and the midpoint of the axis (*M*) refers to the overall sample average across domains. PEAA = perceived expectations for active aging. PH = physical health. MH = mental health. SE = Social engagement. All changes in the mean level were significant at *p* < .001, except for preparation in the physical health and mental health domains (*p* = .019 and .011, respectively, which was not significant at the adjusted *p*-level of .01)

As shown in Fig. 1, the mean level of all of the outcome variables was lower in 2022 than in 2016.^1^ Specifically, for PEAA, repeated-measures ANOVA showed a significant main effect of time across domains, *F* (1, 626) = 36.453, *p* < 0.001, η_p_^2^ = 0.055, but the interaction with age was not significant, *F* (4, 626) = 0.730, *p* = 0.572. The Wald test confirmed these findings: Significant mean differences emerged across the domains of physical health, *W* = 40.60, *p* < 0.001, mental health, *W* = 42.49, *p* < 0.001, and social engagement, *W* = 34.08, *p* < 0.001. We observed a similar pattern for positive age stereotypes: Time had a significant main effect across domains, *F* (1, 609) = 37.618, *p* < 0.001, *η*_p_^2^ = 0.058, whereas age differences were not significant, *F* (4, 609) = 1.407, *p* = 0.230. Likewise, the Wald test confirmed the significant mean differences in the domains of physical health, *W* = 40.84, *p* < 0.001, mental health, *W* = 16.16, *p* < 0.001, and social engagement, *W* = 22.41, *p* < 0.001. Regarding preparation, time had a significant main effect, *F* (1, 626) = 23.071, *p* < 0.001, *η*_p_^2^ = 0.036, and the interaction with age was again not significant, *F* (4, 626) = 2.996, *p* = 0.018. However, according to the Wald test, the mean difference was significant only in the social engagement domain, *W* = 16.38, *p* < 0.001. Therefore, Hypothesis 1 was supported for aging-related beliefs and partially supported for behaviors. In supplementary analyses with weighted data, mean differences in preparation were significant across domains; other comparisons were consistent with the main findings. With respect to age stereotypes, the decrease in mean levels in weighted data were larger in emerging adults (aged 16–24) compared to those in young-old adults (aged 55–69; *p* < 0.01).

### Residual change analyses

Results from the model with all predictors in the equation are presented in Table 2. Full results from different regression blocks are shown in Supplements 6–8. We will first summarize the effects of control variables across models. Women reported more positive change in PEAA in the social engagement domain than men did. General health was most consistently related to the residual growth in preparation in the physical health domain, while its effects on the residual growth in PEAA were significant or approached significance in the social engagement domain. Age was associated with residual growth in preparation in the mental health domain and positive age stereotypes in the social engagement domain. In the same domain, subjective socioeconomic status had effects on the residual growth in preparation and positive age stereotypes that were significant or approached significance. Lastly, no significant interaction effects between general health and age for any of the outcomes emerged (not shown in tables).

**Table 2:** Residual change analyses results: predicting PEAA, age stereotypes, and preparation in 2022

Predictor	PEAA			Positive age stereotypes			Preparation		
	PH	MH	SE	PH	MH	SE	PH	MH	SE
*Control variables*									
Outcome in 2016	0.23** (0.05)	0.19** (0.05)	0.32** (0.04)	0.27** (0.04)	0.22** (0.04)	0.20** (0.05)	0.35** (0.04)	0.32** (0.04)	0.26** (0.05)
Age	0.00 (0.00)	0.00 (0.00)	− 0.01† (0.00)	0.00 (0.00)	0.01† (0.00)	0.01* (0.00)	0.01 (0.00)	0.02** (0.00)	0.01 (0.00)
Female	0.23 (0.15)	0.15 (0.15)	0.44* (0.14)	0.11 (0.10)	0.07 (0.10)	0.07 (0.10)	0.29† (0.13)	0.05 (0.12)	0.16 (0.13)
Subjective SES	0.02 (0.09)	0.03 (0.09)	0.01 (0.09)	− 0.03 (0.06)	− 0.01 (0.06)	0.17† (0.07)	0.11 (0.07)	0.12 (0.07)	0.22† (0.09)
Partner	0.15 (0.15)	0.16 (0.15)	0.20 (0.14)	0.16 (0.11)	0.02 (0.09)	− 0.13 (0.10)	0.06 (0.13)	0.01 (0.11)	0.02 (0.13)
General health	0.08 (0.10)	0.16 (0.10)	0.25† (0.10)	0.13 (0.07)	0.08 (0.07)	0.05 (0.07)	0.40** (0.09)	0.07 (0.09)	0.16 (0.09)
*Socioeconomic predictors*									
Education	0.02 (0.03)	0.02 (0.03)	0.01 (0.03)	0.00 (0.02)	0.00 (0.02)	− 0.02 (0.03)	0.03 (0.03)	0.04 (0.03)	− 0.01 (0.03)
Telework^a^	− 0.01 (0.33)	0.18 (0.33)	0.35 (0.31)	− 0.01 (0.22)	0.16 (0.20)	0.16 (0.24)	0.15 (0.27)	0.74* (0.26)	− 0.30 (0.31)
Most disrupted^a^	− 0.08 (0.34)	0.10 (0.34)	0.42 (0.33)	− 0.17 (0.23)	0.19 (0.21)	0.05 (0.25)	0.42 (0.28)	0.89** (0.25)	0.00 (0.33)
Healthcare^a^	0.30 (0.40)	0.31 (0.38)	0.14 (0.39)	− 0.07 (0.27)	− 0.06 (0.26)	− 0.20 (0.28)	− 0.24 (0.33)	0.77† (0.32)	0.12 (0.36)
Manufacturing^a^	− 0.05 (0.38)	− 0.18 (0.39)	0.29 (0.33)	− 0.16 (0.24)	0.04 (0.22)	− 0.13 (0.24)	0.17 (0.32)	0.52 (0.28)	− 0.32 (0.37)
Income difference	0.05 (0.07)	0.00 (0.07)	− 0.08 (0.06)	0.02 (0.05)	0.02 (0.05)	0.08† (0.05)	0.02 (0.06)	− 0.01 (0.06)	0.06 (0.05)
Children at home	− 0.40* (0.18)	− 0.29† (0.18)	− 0.49** (0.18)	− 0.04 (0.13)	0.08 (0.13)	0.04 (0.14)	− 0.14 (0.16)	− 0.06 (0.17)	− 0.25 (0.17)
*Political attitudes*									
Populism	− 0.18 (0.15)	0.04 (0.16)	− 0.35* (0.14)	0.01 (0.13)	0.07 (0.10)	− 0.03 (0.12)	− 0.06 (0.15)	0.27 (0.14)	− 0.06 (0.15)
No party preference^b^	− 0.40 (0.65)	− 0.47 (0.66)	− 0.17 (0.56)	0.10 (0.48)	0.29 (0.42)	0.15 (0.51)	− 0.05 (0.54)	− 1.21† (0.49)	− 0.26 (0.63)
Non-centrist party^b^	− 0.12 (0.91)	− 0.51 (0.94)	− 0.94 (0.89)	− 0.11 (0.66)	− 0.51 (0.61)	− 1.14 (0.73)	0.14 (0.79)	− 1.09 (0.76)	− 0.67 (0.86)
Strength of support	− 0.05 (0.18)	− 0.09 (0.19)	0.02 (0.16)	0.00 (0.14)	0.08 (0.12)	0.01 (0.15)	− 0.01 (0.15)	− 0.33† (0.14)	− 0.13 (0.18)
Non-centrist party x support	− 0.02 (0.26)	0.10 (0.27)	0.25 (0.25)	0.06 (0.18)	0.17 (0.17)	0.38 (0.20)	− 0.06 (0.23)	0.30 (0.21)	0.25 (0.24)
*R*^2^	.089	.066	.204	.091	.077	.141	.255	.321	.188
Δ*R*^2^	.020	.010	.040	.004	.014	.038	.014	.036	.027

*N* = 2,007. Cells show unstandardized regression coefficients with standard errors in parentheses. Δ*R*^2^ refers to the difference from the baseline model (model with control variables only). PEAA = perceived expectations for active aging. PH = physical health domain. MH = mental health domain. SE = social engagement domain. SES = subjective socioeconomic status. Income difference was standardized

^a^Reference group: occupations less disrupted by the pandemic. ^b^Reference group: centrist-party preference

^†^*p* < .10 *p < .05. **p < .01. ***p < .001. (for planned significance tests)

^†^*p* < .05. *p < .01. **p < .001. (for post-hoc significance tests, including those for control variables)

Next, we will describe the effects relevant to our hypotheses. In the models with socioeconomic predictors (Model 1), having children in the household (vs. not) was associated with more negative change in PEAA in the physical health, *B*(*SE*) = − 0.40 (0.18), *p* = 0.028, β = − 0.11, and social engagement domains, *B*(*SE*) = − 0.47 (0.18), *p* = 0.009, β = − 0.11. In the mental health domain, this effect approached significance, *B*(*SE*) = − 0.30 (0.18), *p* = 0.084, β = − 0.08. The final model (i.e., with all predictors in the equation) confirmed these associations (see Table 2). There were no significant domain differences. For residual change in positive age stereotypes, no significant effects of socioeconomic predictors emerged, except for the expectedly positive effect of income difference, which approached significance, in the social engagement domain (only in the full model; see Table 2).

Regarding preparation, unexpectedly, individuals working in occupations disrupted by the pandemic reported more positive change in preparation in the mental health domain than those working in less disrupted occupations. Specifically, residual growth in preparation in this domain was significantly predicted by working in the most disrupted occupations, *B*(*SE*) = 0.89 (0.26), *p* < 0.001, β = 0.25 (significantly more positive than in the social engagement domain, *p* = 0.002), while the effects of teleworking, *B*(*SE*) = 0.62 (0.26), *p* = 0.017, β = 0.17, and working in healthcare, *B*(*SE*) = 0.71 (0.32), *p* = 0.026, β = 0.14, approached significance according to our significance threshold for unexpected effects. In the final model, these findings remained unchanged (see Table 2). Educational attainment had no significant effects on any of the outcomes. Thus, with respect to socioeconomic predictors, only Hypothesis 3 received some support for PEAA as an outcome.

Furthermore, a greater affinity for populism significantly predicted more negative change in PEAA in the social engagement domain both in Model 2 (with political attitudes), *B*(*SE*) = − 0.32 (0.12), *p* = 0.008, β = − 0.13, and in the final model. No other significant effects of political attitudes were found. Therefore, we obtained partial support only for Hypothesis 6 regarding PEAA.

The results of the supplementary analyses of age differences in the predictor effects (not shown in tables) were largely consistent with the main findings. One difference was that among adults aged under 55, a stronger support for centrist political parties predicted more negative residual change in preparation in the mental health domain, *B*(*SE*) = − 0.66 (0.25), *p* = 0.008, β = − 0.72. This effect approached significance in the entire sample (see Table 2) but was close to zero in older adults. In addition, self-rated health sometimes appeared a more potent predictor of age-related beliefs and behaviors in older than in middle-aged and younger adults. Moreover, the results of weighted regression analyses were consistent with the unweighted ones (see Supplement 9). Finally, across domains, there were no significant differences in the associations between predictors and outcomes at T_1_ between the participants who remained at T_2_ and those who dropped out. Thus, the missing at random assumption appeared to be fulfilled.

## Discussion

In this study, we examined changes in aging-related beliefs and behaviors during the COVID-19 pandemic and in whom this change was more positive or negative. On average, across the domains of physical health, mental health, and social engagement, German respondents perceived lower expectations for active aging and reported less positive age stereotypes in 2022 than in 2016. Aside from a minor decrease in the social engagement domain, preparation for age-related changes did not change. Actually, the magnitude of negative change may be underestimated, as the supplementary weighted analyses suggested a more pronounced mean change, whereas the dropout analyses revealed lower levels of positive age stereotypes and preparation at T_1_ in those who dropped out of the survey. Against the backdrop of far-reaching policy restrictions and pervasive threats to public health, older adults were continuously portrayed as vulnerable and in need of protection, and appeals for solidarity and responsibility prevailed over those for active aging (Ellerich-Groppe et al. 2021; Schönweitz et al. 2024). Negative domain-general changes in PEAA and age stereotypes seemed to reflect these circumstances and the widespread old-age narrative during the pandemic. These findings on mean change also corroborate the dynamic nature and contextual embeddedness of views on aging (cf. Kornadt and Rothermund 2011; Radoš et al. 2025). In contrast, the continuity of health-related preparation—and, for some individuals, its increase in the mental health domain—suggested that maintaining such behaviors was an adaptive response to the heightened health-related risks and restrictions during the pandemic (cf. Kim-Knauss et al. 2022). The salient vulnerability narrative may have reinforced the relevance of health-related preparation. However, because pandemic-control measures had already eased in Germany by the time of our second measurement, preparation—as well as age stereotypes and PEAA—could have been more affected at the earlier stages of the pandemic (cf. Herbolsheimer et al. 2024; Wahl et al. 2022).

For residual change analyses, we expected greater decreases across outcomes among participants with lower education, with children at home, with work-related disruption, and with reduced income during the pandemic as well as among those with non-centrist (i.e., oppositional) political views and populist leanings. We found limited support for these hypotheses. Regarding PEAA, the participants living with children reported a greater decrease across domains than those without children. The policy response to the pandemic altered family household dynamics by imposing additional stressful responsibilities on parents, while the recognition of their care burden was limited and partly dependent on their employment status (Li et al. 2022; Zagel and Struffolino 2026). Thus, individuals in charge of children may have felt unrecognized and perceived the broader society or policymakers to be unconcerned about their well-being and staying active. Furthermore, a stronger affinity for populism predicted a greater reduction in PEAA in the social engagement domain. In Germany, the importance and safety of immunization were continually emphasized. Moreover, during the peak incidence periods, proof of vaccination, certificate of recovery from the COVID-19, or a negative test was required to access public venues and facilities. Many individuals with populist beliefs resisted this narrative (Ehrke et al. 2023) and consequently faced a temporary exclusion from certain spheres of public life.

None of the focal predictors significantly predicted change in positive age stereotypes, but some of the control variables did. Positive age stereotypes in the social engagement domain—in which age differences had not previously been found (Kornadt and Rothermund 2011)—decreased less among older participants, perhaps because many older adults remained socially engaged (Simonson and Kelle 2023). In the weighted analyses, negative change in age stereotypes appeared more pronounced among emerging adults than in young-old adults. This may partly reflect younger individuals’ heightened concern about older adults’ consumption of shared resources during the pandemic (cf. North & Fiske 2013). As part of supplementary analyses, we found an isolated effect of migration background in the mental health domain (*p* < 0.001), whereby participants with (vs. without) migration background had a more pronounced negative change in age stereotypes. This finding may reflect cultural variation in such beliefs. The effects of other control variables replicated pre-pandemic findings on the corresponding aging-related constructs (Kim-Knauss et al. 2022; Kornadt and Rothermund 2011; Pavlova and Silbereisen 2012; Pavlova et al. 2023).

Unexpectedly, we found increased preparation in the mental health domain among those working in occupations affected by the pandemic, including healthcare workers. The psychological toll likely accompanied occupational disruption; indeed, mental health in Germany continuously declined from 2018 to 2022 (Patzina et al. 2025). Thus, individuals experiencing work-related disruption may have been more aware of mental health issues and, accordingly, more motivated to prevent or address them. Alternatively, this finding may reflect adaptation to new cognitively demanding demands (e.g., learning digital tools after switching to remote work), which may have also been perceived as supporting the long-term maintenance of mental fitness.

Using unique domain-specific data for three distinct categories of aging-related beliefs and behaviors in 2016 and 2022 was the central strength of our study. Against the backdrop of previous longitudinal studies that focused mostly on older adults (e.g.,Seifert 2021; Wahl et al. 2022), our sample’s broad age range enabled us to explore whether aging-related beliefs changed across adult age groups. Furthermore, our sample was nationally representative, and supplementary weighted analyses supported the generalizability of our findings to the German population. However, residual change analyses based on two measurement occasions cannot fully capture the trajectory of change and may not completely account for within-person covariation or distinguish within-person change from baseline between-person differences. The large longitudinal dropout was a limitation. However, supplementary sensitivity analyses suggested that the missing at random assumption was met by our data, therefore full information estimation methods and including relevant covariates into the models were adequate approaches of dealing with missingness. While we acknowledge that demographic and other historical factors, as well as random fluctuations, may contribute to changes in age-related beliefs (cf. Wolff et al. 2018), the COVID-19 pandemic was the most disruptive and prolonged event in the period under examination. In fact, the negative change in our outcomes opposes both the pre-pandemic historical trends in Germany and the well-known positive age-graded change in age stereotypes (e.g., Kornadt and Rothermund 2011) and aligns with previous research that noted a trend toward less positive aging-related beliefs and perceptions in Germany during the pandemic (Wahl et al. 2022; Wettstein et al. 2023a). Future studies should determine whether the observed decline in positive aging-related beliefs (cf. Wettstein et al. 2023a) continued, slowed down, or reversed, and identify further trend correlates—preferably with latent modeling of change. Lastly, several constructs were assessed with few or single items: Future research would benefit from more comprehensive measurements as well as the assessment of domains not included in this study (Kornadt and Rothermund 2011, 2014).

Our findings clearly show that aging-related beliefs worsened during the pandemic and thus serve as a cautionary tale about the risks of portraying older adults in a negative, though seemingly benevolent, way. During health crises, the representation of older age should balance facts, intent (e.g., mitigating older adults’ health risk; Molani et al. 2022), and positive images of older age. In the post-pandemic era, policymakers should consider advancing prevention, preparedness, and response planning in a way that would not inadvertently marginalize vulnerable populations. This may also entail a greater scrutiny of media depictions of older adults. Furthermore, renewed efforts to promote active aging could, in addition to middle-aged and older adults, also target groups that experienced disproportionate pandemic-related pressures (e.g., parents, emerging adults), to lower the overall risk of internalizing negative age-related beliefs. The stability of preparatory behaviors may become a foundation on which to build such efforts.

In sum, this study provided evidence that PEAA and descriptive age stereotypes are context-dependent and can shift significantly in a relatively short period. More specifically, beliefs about aging in German adults of all ages appeared to worsen between 2016 and 2022, whereas preparation for age-related changes remained stable. Showcasing older adults as vulnerable and promoting withdrawal from the public sphere during the pandemic may have contributed to a reduction in positive aging-related beliefs. Still, the fact that German adults continued to prepare for age-related changes suggests that such behaviors were adaptive amid the challenges posed by and limitations imposed by the COVID-19 pandemic.

## Supplementary Information

Below is the link to the electronic supplementary material.

Supplementary file1 (DOCX 96 KB)

## Data Availability

We used data from the Innovation Sample of the German Socio-Economic Panel, which included our own data module approved and administered by the SOEP. To access the data, the German data protection laws require all users to sign a data distribution contract with the Deutsches Institut für Wirtschaftsforschung (DIW). Further inquiries can be directed to the DIW https://www.diw.de/de/diw_01.c.615551.de/forschungsbasierte_infrastruktureinrichtung__sozio-oekonomisches_panel__soep.html

## Footnotes

1 To check whether change was specific to aging-related beliefs and behaviors, we repeated our analyses on well-being and personality measures (i.e., constructs unrelated to aging). Only sense of meaning showed a significant decline, considerably smaller than that observed in our outcomes. Therefore, the decline appeared to be specific to aging-related variables.
