# Supplementary material for: Did aging-related beliefs and behaviors change during the COVID-19 pandemic?
Source: Eur J Ageing. 2026 Jul 7;23(1):31. doi: 10.1007/s10433-026-00929-6 (PMC13341994; doi:10.1007/s10433-026-00929-6)
Supplement: Supplementary file 1 — Supplementary file1 (DOCX 96 KB) [file 10433_2026_929_MOESM1_ESM.docx]

Supplement 1. Categorization of occupations by sector and type of disruption by the COVID-19 pandemic

| *Less disrupted by the pandemic* | *Teleworking* | *Most disrupted by the pandemic* | *Healthcare* | *Manufacturing* |
| --- | --- | --- | --- | --- |
| Architectural and engineering activities, technical testing and analysis  Civil engineering  Collection, purification, and distribution of water  Construction of buildings  Crop and animal production, hunting and related service  Electricity, gas, steam, and hot water supply  Forestry and logging  Other mining and quarrying  Private households with employed persons  Programming and broadcasting  Publishing activities  Repair of computers and personal or household goods  Security and investigation  Sewerage  Services to buildings and landscape activities  Specialized construction  Telecommunications  Waste collection, treatment, and disposal | Activities auxiliary to financial and insurance services  Activities of head offices, management consultancy  Advertisement and market research  Computer programming, consultancy, and related  Education  Employment-related  Financial service, except insurance and pension funding  Information service  Insurance, reinsurance, pension funding  Legal and accounting  Office administrative, office and other business support  Other professional, scientific, and technical activities  Public administration and defense, compulsory social security  Real estate, property  Rental and leasing  Research and development | Air transport  Accommodation  Activities of membership organizations  Creative, arts and entertainment  Food and beverage service  Land transport and transport via pipelines  Libraries, archives, museums, and other cultural activities  Motion picture, video, and television program production, movie theaters, sound  Other personal service activities  Postal and courier activities  Retail trade except of motor vehicles or motorcycles  Social work activities without accommodation  Sports and amusement and recreation  Travel agency, tour operator and other reservation service and related  Warehousing and support activities for transportation  Wholesale and retail trade and repair of motor vehicles or motorcycles  Wholesale trade except of motor vehicles or motorcycles | Health service  Residential care activities | Basic metals  Basic pharmaceutical products  Beverages  Chemicals and chemical products  Computer, electronic, and optical products  Electrical equipment  Fabricated metals, ex. machinery and equipment  Food products  Furniture  Machinery and equipment NEC  Motor vehicles and trailers  Other manufacturing  Other non-metallic products  Other transport equipment  Printing and reproduction of recorded media  Pulp and paper products  Rubber and plastic products  Textiles  Wood (except furniture) |

*Note*. Occupations labeled most disrupted were those in which individuals either stopped working entirely, worked under inconvenient restrictions, or worked significantly more during the pandemic, whereas less disrupted occupations underwent minimal or no change. Occupations that include health and residential care services were highly disrupted occupations within the healthcare sector. Teleworking occupations were characterized by a transition to predominantly or fully remote (online) work. Manufacturing was an independent category, as individuals with these occupations experienced the highest increase in short-time work (Engstler et al., 2023).

Supplement 2. Descriptive statistics: outcomes

| Variable | T_1_ (2016)  *M* (*SD*) | | T_2_ (2022)  *M* (*SD*) |
| --- | --- | --- | --- |
| PEAA: physical health | | 5.1 (1.7) | 4.6 (1.7) |
| PEAA: mental health | | 5.4 (1.6) | 5.0 (1.7) |
| PEAA: social engagement | | 3.8 (1.8) | 3.5 (1.8) |
| Positive age stereotypes: physical health | | 4.2 (1.2) | 3.9 (1.2) |
| Positive age stereotypes: mental health | | 4.5 (1.2) | 4.3 (1.1) |
| Positive age stereotypes: social engagement | | 4.5 (1.3) | 4.3 (1.2) |
| Preparation for age-related changes: physical health | | 5.0 (1.7) | 4.9 (1.7) |
| Preparation for age-related changes: mental health | | 5.5 (1.6) | 5.3 (1.6) |
| Preparation for age-related changes: social engagement | | 5.2 (1.6) | 5.0 (1.6) |

*Note.* *N_T1_* = 2,007, *N_T2_* = 733. PEAA = perceived expectations for active aging. Item missings did not exceed 2.2%. All items had the same value range (1–7).

Supplement 3. Bivariate correlations between predictor and outcome variables at baseline

| Variable | 1 | 2 | 3 | 4 | 5 | 6 | 7 | 8 | 9 | 10 | 11 | 12 | 13 | 14 | 15 | 16 |
| --- | --- | --- | --- | --- | --- | --- | --- | --- | --- | --- | --- | --- | --- | --- | --- | --- |
| 1. PEAA: PH | — | .73** | .40** | .10** | .07** | .11** | .23** | .19** | .20** | .00 | .05 | .00 | .01 | .03 | .01 | .02 |
| 2. PEAA: MH |  | — | .40** | .08** | .11** | .11** | .18** | .22** | .20** | .02 | .04 | .05 | -.00 | .02 | .00 | .05 |
| 3. PEAA: SE |  |  | — | .06** | .07** | .12** | .16** | .19** | .27** | .08** | .03 | .02 | .01 | -.05 | .05 | .08** |
| 4. Age stereotypes: PH |  |  |  | — | .71** | .53** | .19** | .17** | .21** | -.02 | .09* | -.03 | -.10** | -.06 | -.11** | .06 |
| 5. Age stereotypes: MH |  |  |  |  | — | .54** | .15** | .16** | .21** | .01 | .04 | -.04 | -.09** | -.04 | -.09* | .02 |
| 6. Age stereotypes: SE |  |  |  |  |  | — | .23** | .24** | .27** | .08** | -.02 | -.05 | -.13** | -.08* | -.09* | .07* |
| 7. Preparation: PH |  |  |  |  |  |  | — | .47** | .59** | .07** | .09* | .07 | -.17** | -.04 | -.07 | .13* |
| 8. Preparation: MH |  |  |  |  |  |  |  | — | .49** | .11** | .07 | -.02 | -.24** | -.01 | -.09* | .15* |
| 9. Preparation: SE |  |  |  |  |  |  |  |  | — | .10* | .08* | .02 | -.17** | -.02 | -.05 | .01** |
| 10. Education |  |  |  |  |  |  |  |  |  | — | .00 | .09* | .13** | -.36** | .13** | .17** |
| 11. Occupation |  |  |  |  |  |  |  |  |  |  | — | -.04 | .18 | .07 | .01 | .20 |
| 12. Income change |  |  |  |  |  |  |  |  |  |  |  | — | -.06 | .00 | .01 | -.02 |
| 13. Children at home |  |  |  |  |  |  |  |  |  |  |  |  | — | -.15** | .12** | -.08* |
| 14. Affinity for populism |  |  |  |  |  |  |  |  |  |  |  |  |  | — | .08 | -.22** |
| 15. Party preference |  |  |  |  |  |  |  |  |  |  |  |  |  |  | — | .08 |
| 16. Political support |  |  |  |  |  |  |  |  |  |  |  |  |  |  |  | — |

*Note.* PEAA = perceived expectations for active aging. PH = physical health. MH = mental health. SE = social engagement. Outcomes at baseline (T_1_) are shown. Measurements of predictor variables were taken from data collection waves between T_1_ and T_2_ (2019–2022).

**p* < .05. ***p* < .01.

Supplement 4. Bivariate correlations between predictor and outcome variables at T_2_

| Variable | 1 | 2 | 3 | 4 | 5 | 6 | 7 | 8 | 9 | 10 | 11 | 12 | 13 | 14 | 15 | 16 |
| --- | --- | --- | --- | --- | --- | --- | --- | --- | --- | --- | --- | --- | --- | --- | --- | --- |
| 1. PEAA: PH | — | .73** | .46** | .13** | .10** | .12** | .16** | .15** | .15** | .07 | -.06 | .05 | -.06 | -.07 | -.10 | .07 |
| 2. PEAA: MH |  | — | .48** | .13** | .12** | .13** | .12** | .19** | .14** | .05 | -.01 | .03 | -.07 | .04 | -.06 | .06 |
| 3. PEAA: SE |  |  | — | .12** | .14** | .17** | .16** | .14** | .26** | .13** | .04 | -.00 | -.03 | -.19** | -.05 | .14** |
| 4. Age stereotypes: PH |  |  |  | — | .57** | .52** | .18** | .17** | .23** | .01 | .03 | .02 | -.01 | -.01 | -.05 | -.00 |
| 5. Age stereotypes: MH |  |  |  |  | — | .56** | .15** | .24** | .21** | .01 | .04 | .01 | -.03 | .00 | -.02 | .04 |
| 6. Age stereotypes: SE |  |  |  |  |  | — | .23** | .25** | .33** | .07 | -.03 | .06 | -.08* | -.02 | -.04 | .05 |
| 7. Preparation: PH |  |  |  |  |  |  | — | .36** | .44** | .14** | .07 | .07 | -.09* | -.07 | -.04 | .06 |
| 8. Preparation: MH |  |  |  |  |  |  |  | — | .38** | .10** | .12* | -.01 | -.22** | .09 | -.09 | .07 |
| 9. Preparation: SE |  |  |  |  |  |  |  |  | — | .09* | .04 | .05 | -.11** | .01 | -01 | .03 |
| 10. Education |  |  |  |  |  |  |  |  |  | — | -.01 | .14** | .11** | -.35** | .08 | .16** |
| 11. Occupation |  |  |  |  |  |  |  |  |  |  | — | -.03 | .01 | .01 | -.01 | -.01 |
| 12. Income change |  |  |  |  |  |  |  |  |  |  |  | — | -.07 | .02 | -.01 | .02 |
| 13. Children at home |  |  |  |  |  |  |  |  |  |  |  |  | — | -.19** | .12* | -.02 |
| 14. Affinity for populism |  |  |  |  |  |  |  |  |  |  |  |  |  | — | .08 | -.21** |
| 15. Party preference |  |  |  |  |  |  |  |  |  |  |  |  |  |  | — | .07 |
| 16. Political support |  |  |  |  |  |  |  |  |  |  |  |  |  |  |  | — |

*Note.* PEAA = perceived expectations for active aging. PH = physical health. MH = mental health. SE = social engagement. Outcomes at T_2_ are shown. Measurements of predictor variables were taken from data collection waves between T_1_ and T_2_ (2019–2022).

**p* < .05. ***p* < .01.

Supplement 5. Outcome variable items.

| Outcome | Domain | Item in English | Item in German |
| --- | --- | --- | --- |
| *Introduction to PEAA*  English: “In the following, we want to ask you about aging and social expectations. Please think of your everyday life. In our society, we often face certain expectations of other people. The following questions relate to those expectations”  German: “Im Folgenden möchten wir uns mit Ihnen über das Altern und gesellschaftliche Erwartungen unterhalten. Denken Sie nun an Ihren Alltag. In unserer Gesellschaft sieht man sich oft bestimmten Erwartungen ausgesetzt. Die folgenden Fragen beziehen sich auf solche Erwartungen ” | | | |
| Perceived expectations for active aging (PEAA) | Physical health | *It is expected of me to keep myself physically fit* | *Es wird von mir erwartet, mich körperlich fit zu halten* |
|  | Mental health | *It is expected of me to keep myself mentally fit* | *Es wird von mir erwartet, mich geistig fit zu halten* |
|  | Social engagement | *People have high expectations that I get involved in social and non-profit-making activities* | *Die Erwartungen sind hoch, dass ich mich für soziale oder gemeinnützige Zwecke einsetze* |
| *Introduction to age stereotypes*  English: “Now we are interested in your view of older people”  German: “Jetzt möchten wir gerne von Ihnen wissen, welches Bild Sie persönlich von älteren Menschen haben” | | | |
| Positive age stereotypes | Physical health | *Older people are healthy and physically fit* | *Ältere Menschen sind gesund und körperlich fit* |
|  | Mental health | *Older people are mentally fit* | *Ältere Menschen sind geistig fit* |
|  | Social engagement | *Older people have a lot of energy and power for meaningful activities, e.g. hobbies or voluntary activities* | *Ältere Menschen besitzen viel Energie und Kraft, um sinnvolle Aktivitäten auszuführen (z. B. Hobbies oder ehrenamtliche Aufgaben)* |
| *Introduction to preparation*  English: “Now we are interested in how you prepare yourself for aging and to what extent you prepare yourself for age-related changes”  German: “Nun interessiert uns, wie Sie sich auf ihr Alter vorbereiten und in welchem Umfang Sie Vorsorge für mögliche altersbedingte Veränderungen treffen” | | | |
| Preparation for age-related changes | Physical health | *I actively take care to keep my physical fitness in older age, e.g. by regularly working out or avoiding health risks* | *Ich sorge aktiv dafür, meine körperliche Fitness im höheren Alter zu erhalten (z.B. durch regelmäßigen Sport und die Vermeidung von Gesundheitsrisiken)* |
|  | Mental health | *I actively take care to keep my mental fitness in older age, e.g. by mental activity in solving crossword puzzles or reading books and magazines* | *Ich sorge aktiv dafür, meine geistige Fitness im höheren Alter zu erhalten (z.B. durch geistige Betätigung wie Kreuzworträtsel lösen oder Bücher und Zeitschriften lesen)* |
|  | Social engagement | *I actively take care to be able to perform meaningful activities in older age, e.g. by caring about my hobbies or adopting tasks in my family circle, neighborhood or*  *community* | *Ich sorge aktiv dafür, auch im hohen Alter viele sinnvolle Aktivitäten ausführen zu können (z.B. pflege ich meine Hobbies oder übernehme Aufgaben im Familienkreis, in der Nachbarschaft oder in der Gemeinde)* |

Supplement 6. Residual change analyses results: Predicting PEAA in 2022

| Predictor | Model 1 | | | Model 2 | | | Model 3 | | |
| --- | --- | --- | --- | --- | --- | --- | --- | --- | --- |
|  | PH | MH | SE | PH | MH | SE | PH | MH | SE |
| *Control variables* |  |  |  |  |  |  |  |  |  |
| PEAA in 2016 | 0.23**  (0.05) | 0.20**  (0.05) | 0.33**  (0.04) | 0.23**  (0.05) | 0.19**  (0.05) | 0.31**  (0.04) | 0.23**  (0.05) | 0.19**  (0.05) | 0.32**  (0.04) |
| Age | -0.00  (0.00) | 0.00  (0.00) | -0.01†  (0.00) | 0.00  (0.00) | 0.00  (0.00) | -0.00  (0.00) | -0.00  (0.00) | -0.00  (0.00) | -0.01†  (0.00) |
| Female | 0.24  (0.15) | 0.15  (0.15) | 0.43*  (0.14) | 0.27†  (0.13) | 0.24  (0.13) | 0.44**  (0.12) | 0.23  (0.15) | 0.15  (0.15) | 0.44*  (0.14) |
| Subjective socioeconomic status | 0.01  (0.09) | 0.03  (0.09) | -0.02  (0.08) | 0.07  (0.08) | 0.09  (0.08) | 0.05  (0.08) | 0.02  (0.09) | 0.03  (0.09) | 0.01  (0.09) |
| Cohabiting with a partner | 0.17  (0.15) | 0.17  (0.15) | 0.22  (0.14) | 0.05  (0.14) | 0.08  (0.14) | 0.09  (0.13) | 0.15  (0.15) | 0.16  (0.15) | 0.20  (0.14) |
| General health | 0.12  (0.09) | 0.15  (0.09) | 0.33*  (0.10) | 0.08  (0.10) | 0.13  (0.10) | 0.23†  (0.10) | 0.08  (0.10) | 0.16  (0.10) | 0.25†  (0.10) |
| *Socioeconomic predictors* |  |  |  |  |  |  |  |  |  |
| Educational attainment | 0.03  (0.03) | 0.01  (0.03) | 0.05  (0.03) |  |  |  | 0.02  (0.03) | 0.02  (0.03) | 0.01  (0.03) |
| Teleworking occupations^a^ | 0.01  (0.33) | 0.17  (0.32) | 0.44  (0.31) |  |  |  | -0.01  (0.33) | 0.18  (0.33) | 0.35  (0.31) |
| Most disrupted occupations^a^ | -0.11  (0.34) | 0.11  (0.33) | 0.37  (0.33) |  |  |  | -0.08  (0.34) | 0.10  (0.34) | 0.42  (0.33) |
| Healthcare^a^ | 0.29  (0.40) | 0.30  (0.38) | 0.21  (0.39) |  |  |  | 0.30  (0.40) | 0.31  (0.38) | 0.14  (0.39) |
| Manufacturing occupations^a^ | -0.09  (0.38) | -0.19  (0.38) | 0.24  (0.33) |  |  |  | -0.05  (0.38) | -0.18  (0.39) | 0.29  (0.33) |
| Income difference 2022-2016^b^ | 0.05  (0.07) | 0.00  (0.07) | -0.10†  (0.06) |  |  |  | 0.05  (0.07) | 0.00  (0.07) | -0.08  (0.06) |
| Children in the household | -0.40*  (0.18) | -0.30†  (0.18) | -0.47**  (0.18) |  |  |  | -0.40*  (0.18) | -0.29†  (0.18) | -0.49**  (0.18) |
| *Political attitudes* |  |  |  |  |  |  |  |  |  |
| Affinity for populism |  |  |  | -0.16  (0.14) | 0.11  (0.14) | -0.32**  (0.12) | -0.18  (0.15) | 0.04  (0.16) | -0.35*  (0.14) |
| Non-centrist-party preference |  |  |  | -0.18  (0.90) | -0.54  (0.91) | -0.95  (0.88) | -0.12  (0.91) | -0.51  (0.94) | -0.94  (0.89) |
| No party preference^c^ |  |  |  | -0.46  (0.64) | -0.51  (0.64) | -0.21  (0.55) | -0.40  (0.65) | -0.47  (0.66) | -0.17  (0.56) |
| Strength of party support |  |  |  | -0.06  (0.18) | -0.09  (0.18) | 0.00  (0.16) | -0.05  (0.18) | -0.09  (0.19) | 0.02  (0.16) |
| Non-centrist party preference x support strength |  |  |  | 0.01  (0.25) | 0.12  (0.26) | 0.26  (0.25) | -0.02  (0.26) | 0.10  (0.27) | 0.25  (0.25) |
| *R*^2^ | .079 | .063 | .183 | .077 | .062 | .185 | .089 | .066 | .204 |
| *ΔR^2^* | .011 | .009 | .018 | .007 | .003 | .021 | .020 | .010 | .040 |

*Note*. *N* = 2,007. Cells show unstandardized regression coefficients with standard errors in parentheses. PEAA = perceived expectations for active aging. PH = physical health domain. MH = mental health domain. SE = social engagement domain.

^a^ Reference group: occupations less disrupted by the pandemic.

^b^ Standardized in all models.

^c^ Reference group: centrist-party preference.

† *p* < .10 * *p* < .05. ** *p* < .01. *** *p* < .001. (for planned significance tests)

† *p* < .05. * *p* < .01. ** *p* < .001. (for post-hoc significance tests, including those for control variables)

Supplement 7. Residual change analyses results: Predicting positive age stereotypes in 2022

| Predictor | Model 1 | | | Model 2 | | | Model 3 | | |
| --- | --- | --- | --- | --- | --- | --- | --- | --- | --- |
|  | PH | MH | SE | PH | MH | SE | PH | MH | SE |
| *Control variables* |  |  |  |  |  |  |  |  |  |
| Positive age stereotypes in 2016 | 0.27**  (0.04) | 0.22**  (0.04) | 0.21**  (0.05) | 0.26**  (0.04) | 0.21**  (0.04) | 0.20**  (0.05) | 0.27**  (0.04) | 0.22**  (0.04) | 0.20**  (0.05) |
| Age | 0.00  (0.00) | 0.01†  (0.00) | 0.01*  (0.00) | 0.00  (0.00) | 0.01†  (0.00) | 0.01*  (0.00) | 0.00  (0.00) | 0.01†  (0.00) | 0.01*  (0.00) |
| Female | 0.11  (0.10) | 0.06  (0.10) | 0.07  (0.11) | 0.11  (0.09) | 0.08  (0.08) | 0.08  (0.09) | 0.11  (0.10) | 0.07  (0.10) | 0.07  (0.10) |
| Subjective socioeconomic status | -0.03  (0.05) | -0.01  (0.06) | 0.15†  (0.07) | -0.02  (0.05) | -0.02  (0.06) | 0.17*  (0.06) | -0.03  (0.06) | -0.01  (0.06) | 0.17†  (0.07) |
| Cohabiting with a partner | 0.15  (0.11) | 0.01  (0.09) | -0.14  (0.11) | 0.14  (0.10) | 0.03  (0.09) | -0.14  (0.10) | 0.16  (0.11) | 0.02  (0.09) | -0.13  (0.10) |
| General health | 0.12  (0.07) | 0.08  (0.06) | 0.06  (0.07) | 0.11  (0.07) | 0.08  (0.06) | 0.03  (0.07) | 0.13  (0.07) | 0.08  (0.07) | 0.05  (0.07) |
| *Socioeconomic predictors* |  |  |  |  |  |  |  |  |  |
| Educational attainment | -0.00  (0.02) | 0.01  (0.02) | -0.01  (0.03) |  |  |  | -0.00  (0.02) | 0.00  (0.02) | -0.02  (0.03) |
| Teleworking occupations^a^ | -0.02  (0.21) | 0.12  (0.20) | 0.14  (0.24) |  |  |  | -0.01  (0.22) | 0.16  (0.20) | 0.16  (0.24) |
| Most disrupted occupations^a^ | -0.14  (0.23) | 0.20  (0.21) | 0.07  (0.25) |  |  |  | -0.17  (0.23) | 0.19  (0.21) | 0.05  (0.25) |
| Healthcare^a^ | -0.06  (0.26) | -0.06  (0.26) | -0.18  (0.29) |  |  |  | -0.07  (0.27) | -0.06  (0.26) | -0.20  (0.28) |
| Manufacturing occupations^a^ | -0.13  (0.34) | 0.06  (0.21) | -0.13  (0.23) |  |  |  | -0.16  (0.24) | 0.04  (0.22) | -0.13  (0.24) |
| Income difference 2022-2016^b^ | 0.02  (0.05) | 0.02  (0.05) | 0.08  (0.05) |  |  |  | 0.02  (0.05) | 0.02  (0.05) | 0.08†  (0.05) |
| Children in the household | -0.03  (0.13) | 0.08  (0.13) | 0.05  (0.14) |  |  |  | -0.04  (0.13) | 0.08  (0.13) | 0.04  (0.14) |
| *Political attitudes* |  |  |  |  |  |  |  |  |  |
| Affinity for populism |  |  |  | -0.01  (0.11) | 0.03  (0.09) | -0.05  (0.10) | 0.01  (0.13) | 0.07  (0.10) | -0.03  (0.12) |
| Non-centrist-party preference |  |  |  | -0.06  (0.65) | -0.43  (0.60) | -1.09  (0.73) | -0.11  (0.66) | -0.51  (0.61) | -1.14  (0.73) |
| No party preference^c^ |  |  |  | 0.11  (0.48) | 0.30  (0.42) | 0.13  (0.51) | 0.10  (0.48) | 0.29  (0.42) | 0.15  (0.51) |
| Strength of party support |  |  |  | 0.01  (0.14) | 0.08  (0.12) | 0.01  (0.14) | 0.00  (0.14) | 0.08  (0.12) | 0.01  (0.14) |
| Non-centrist party preference x support |  |  |  | 0.04  (0.18) | 0.15  (0.17) | 0.37  (0.20) | 0.06  (0.18) | 0.17  (0.17) | 0.38  (0.20) |
| *R*^2^ | .089 | .071 | .122 | .086 | .068 | .127 | .091 | .077 | .141 |
| *ΔR^2^* | .002 | .008 | .014 | .001 | .006 | .021 | .004 | .014 | .038 |

*Note*. *N* = 2,007. Cells show unstandardized regression coefficients with standard errors in parentheses. PH = physical health domain. MH = mental health domain. SE = social engagement domain.

^a^ Reference group: occupations less disrupted by the pandemic.

^b^ Standardized in all models.

^c^ Reference group: centrist-party preference.

† *p* < .10 * *p* < .05. ** *p* < .01. *** *p* < .001. (for planned significance tests)

† *p* < .05. * *p* < .01. ** *p* < .001. (for post-hoc significance tests, including those for control variables)

Supplement 8. Residual change analyses results: Predicting preparation for age-related changes in 2022

| Predictor | Model 1 | | | Model 2 | | | Model 3 | | |
| --- | --- | --- | --- | --- | --- | --- | --- | --- | --- |
|  | PH | MH | SE | PH | MH | SE | PH | MH | SE |
| *Control variables* |  |  |  |  |  |  |  |  |  |
| Preparation in 2016 | 0.35**  (0.04) | 0.31**  (0.05) | 0.26**  (0.05) | 0.34**  (0.04) | 0.33**  (0.05) | 0.27**  (0.05) | 0.35**  (0.04) | 0.32**  (0.04) | 0.26**  (0.05) |
| Age | 0.01  (0.00) | 0.02**  (0.00) | 0.01  (0.00) | 0.01*  (0.00) | 0.03**  (0.00) | 0.01†  (0.00) | 0.01  (0.00) | 0.02**  (0.00) | 0.01  (0.00) |
| Female | 0.30†  (0.12) | 0.06  (0.12) | 0.16  (0.13) | 0.28†  (0.11) | 0.17  (0.11) | 0.22  (0.11) | 0.29†  (0.12) | 0.05  (0.12) | 0.15  (0.13) |
| Subjective socioeconomic status | 0.11  (0.07) | 0.14†  (0.06) | 0.20†  (0.09) | 0.13  (0.07) | 0.17†  (0.07) | 0.23*  (0.08) | 0.11  (0.07) | 0.12  (0.07) | 0.22†  (0.09) |
| Cohabiting with a partner | 0.07  (0.13) | 0.02  (0.11) | 0.02  (0.13) | 0.03  (0.12) | -0.00  (0.11) | -0.04  (0.12) | 0.06  (0.13) | 0.01  (0.11) | 0.02  (0.13) |
| General health | 0.41**  (0.09) | 0.02  (0.08) | 0.17†  (0.09) | 0.39**  (0.09) | 0.07  (0.09) | 0.15  (0.09) | 0.40**  (0.09) | 0.07  (0.09) | 0.16  (0.09) |
| *Socioeconomic predictors* |  |  |  |  |  |  |  |  |  |
| Educational attainment | 0.03  (0.02) | 0.02  (0.02) | 0.01  (0.03) |  |  |  | 0.03  (0.03) | 0.04  (0.03) | -0.01  (0.03) |
| Teleworking occupations^a^ | 0.16  (0.27) | 0.62  (0.26) | -0.31  (0.32) |  |  |  | 0.15  (0.27) | 0.74**  (0.26) | -0.29  (0.31) |
| Most disrupted occupations^a^ | 0.45  (0.28) | 0.89**  (0.26) | 0.02  (0.37) |  |  |  | 0.42  (0.28) | 0.89**  (0.25) | 0.00  (0.33) |
| Healthcare^a^ | -0.23  (0.33) | 0.71†  (0.32) | 0.14  (0.37) |  |  |  | -0.24  (0.33) | 0.77†  (0.32) | 0.12  (0.36) |
| Manufacturing occupations^a^ | 0.19  (0.31) | 0.52  (0.29) | -0.32  (0.37) |  |  |  | 0.17  (0.32) | 0.52  (0.28) | -0.32  (0.36) |
| Income difference 2022-2016^b^ | 0.02  (0.06) | 0.00  (0.06) | 0.06  (0.05) |  |  |  | 0.02  (0.06) | -0.01  (0.06) | 0.06  (0.05) |
| Children in the household | -0.14  (0.16) | -0.11  (0.17) | -0.25  (0.17) |  |  |  | -0.14  (0.16) | -0.06  (0.17) | -0.25  (0.17) |
| *Political attitudes* |  |  |  |  |  |  |  |  |  |
| Affinity for populism |  |  |  | -0.10  (0.12) | 0.18  (0.12) | 0.02  (0.12) | -0.06  (0.15) | 0.27  (0.14) | -0.06  (0.15) |
| Non-centrist-party preference |  |  |  | 0.08  (0.80) | -1.01  (0.75) | -0.97  (0.84) | 0.14  (0.79) | -1.08  (0.76) | -0.67  (0.86) |
| No party preference^c^ |  |  |  | -0.15  (0.55) | -1.17†  (0.49) | -0.39  (0.62) | -0.05  (0.54) | -1.21†  (0.49) | -0.26  (0.63) |
| Strength of party support |  |  |  | -0.05  (0.16) | -0.32†  (0.14) | -0.16  (0.18) | -0.01  (0.15) | -0.33†  (0.14) | -0.12  (0.18) |
| Non-centrist party preference x support |  |  |  | -0.02  (0.23) | 0.30  (0.21) | 0.33  (0.24) | -0.06  (0.23) | 0.30  (0.21) | 0.25  (0.24) |
| *R*^2^ | .255 | .304 | .181 | .245 | .296 | .183 | .255 | .321 | .188 |
| *ΔR^2^* | .014 | .023 | .018 | .003 | .013 | .009 | .014 | .036 | .027 |

*Note*. *N* = 2,007. Cells show unstandardized regression coefficients with standard errors in parentheses. PH = physical health domain. MH = mental health domain. SE = social engagement domain.

^a^ Reference group: occupations less disrupted by the pandemic.

^b^ Standardized in all models.

^c^ Reference group: centrist-party preference.

† *p* < .10 * *p* < .05. ** *p* < .01. *** *p* < .001. (for planned significance tests)

† *p* < .05. * *p* < .01. ** *p* < .001. (for post-hoc significance tests, including those for control variables)

Supplement 9. Weighted residual change analyses results: Outcomes in 2022

| Predictor | PEAA | | | | | | Positive age stereotypes | | | | | | | | | Preparation | | | | | | | | | |  |
| --- | --- | --- | --- | --- | --- | --- | --- | --- | --- | --- | --- | --- | --- | --- | --- | --- | --- | --- | --- | --- | --- | --- | --- | --- | --- | --- |
|  | PH | | MH | | SE | | PH | | | MH | | | SE | | | PH | | | MH | | | SE | | | |  |
| *Control variables* |  | |  | |  | |  | | |  | | |  | | |  | | |  | | |  | | | |  |
| Outcome in 2016 | 0.25**  (0.05) | | 0.19*  (0.06) | | 0.36**  (0.05) | | 0.30**  (0.05) | | | 0.22**  (0.05) | | | 0.16*  (0.05) | | | 0.34**  (0.05) | | | 0.35**  (0.05) | | | 0.27**  (0.06) | | | |  |
| Age | -0.01  (0.01) | | 0.00  (0.01) | | -0.01†  (0.01) | | 0.01  (0.00) | | | 0.01  (0.00) | | | 0.01*  (0.00) | | | 0.00  (0.01) | | | 0.02**  (0.01) | | | 0.00  (0.01) | | | |  |
| Female | 0.34  (0.18) | | 0.15  (0.18) | | 0.49*  (0.16) | | 0.08  (0.12) | | | -0.00  (0.11) | | | 0.01  (0.12) | | | 0.54**  (0.15) | | | 0.21  (0.13) | | | 0.20  (0.15) | | | |  |
| Subjective SES | 0.01  (0.12) | | -0.02  (0.11) | | 0.01  (0.10) | | -0.05  (0.06) | | | -0.01  (0.06) | | | 0.16†  (0.08) | | | 0.11  (0.08) | | | 0.16†  (0.08) | | | 0.24†  (0.10) | | | |  |
| Partner | 0.22  (0.20) | | 0.08  (0.21) | | 0.40†  (0.16) | | 0.12  (0.13) | | | 0.03  (0.11) | | | -0.10  (0.11) | | | 0.27  (0.15) | | | 0.05  (0.13) | | | 0.23  (0.16) | | | |  |
| General health | 0.10  (0.12) | | 0.20  (0.12) | | 0.21  (0.12) | | 0.19†  (0.09) | | | 0.04  (0.07) | | | 0.06  (0.09) | | | 0.42**  (0.11) | | | 0.01  (0.09) | | | 0.15  (0.10) | | | |  |
| *Socioeconomic predictors* | | | | | | | | | | | | | | | | | | | | | | | | | |  |
| Education | 0.04  (0.04) | | 0.05  (0.04) | | 0.01  (0.04) | | 0.00  (0.03) | | | 0.02  (0.02) | | | -0.00  (0.03) | | | 0.02  (0.04) | | | 0.03  (0.04) | | | -0.02  (0.04) | | | |  |
| Telework^a^ | -0.27  (0.42) | | 0.15  (0.43) | | 0.41  (0.36) | | 0.20  (0.26) | | | 0.37†  (0.21) | | | 0.37  (0.30) | | | 0.03  (0.32) | | | 0.74†  (0.32) | | | -0.47  (0.37) | | | |  |
| Most disrupted^a^ | -0.36  (0.45) | | 0.00  (0.45) | | 0.55  (0.38) | | -0.09  (0.27) | | | 0.34  (0.22) | | | 0.42  (0.29) | | | 0.31  (0.34) | | | 0.75†  (0.31) | | | | | -0.03  (0.38) | |  |
| Healthcare^a^ | -0.28  (0.54) | | 0.11  (0.53) | | -0.10  (0.47) | | 0.18  (0.33) | | | 0.33  (0.33) | | | 0.20  (0.38) | | | -0.52  (0.41) | | | 0.44  (0.40) | | | -0.21  (0.44) | | | |  |
| Manufacturing^a^ | -0.04  (0.46) | | -0.09  (0.46) | | 0.54  (0.38) | | -0.00  (0.30) | | | 0.28  (0.21) | | | 0.22  (0.29) | | | 0.44  (0.37) | | | 0.55  (0.33) | | | -0.36  (0.47) | | | |  |
| Income difference | -0.07  (0.08) | | -0.07  (0.08) | | -0.12  (0.08) | | 0.00  (0.06) | | | -0.01  (0.05) | | | 0.09  (0.06) | | | 0.00  (0.08) | | | 0.03  (0.06) | | | 0.05  (0.07) | | | |  |
| Children at home | -0.50*  (0.25) | | -0.33  (0.26) | | -0.53*  (0.21) | | | 0.13  (0.16) | | | 0.15  (0.15) | | | 0.12  (0.17) | | | -0.42*  (0.20) | | | -0.29  (0.20) | | | -0.27  (0.22) | | | |
| *Political attitudes* | | | | | | | | | | | | | | | | | | | | | | | | | |  |
| Populism | -0.21  (0.18) | | 0.23  (0.19) | | -0.30  (0.17) | | 0.06  (0.15) | | | 0.10  (0.10) | | | -0.00  (0.13) | | | -0.14  (0.18) | | | 0.19  (0.16) | | | -0.07  (0.17) | | | |  |
| No party preference^b^ | -0.26  (0.78) | | -0.08  (0.75) | | 0.45  (0.68) | | 0.00  (0.50) | | | 0.06  (0.42) | | | 0.14  (0.62) | | | -0.19  (0.68) | | | -1.65*  (0.61) | | | -0.50  (0.69) | | | |  |
| Non-centrist-party | 0.24  (1.02) | | -0.04  (1.07) | | -0.21  (1.05) | | -0.04  (0.70) | | | -0.90  (0.63) | | | -1.21  (0.85) | | | 0.60  (1.01) | | | -1.85*  (0.91) | | | -0.57  (0.87) | | | |  |
| Strength of support | -0.06  (0.21) | | -0.01  (0.20) | | 0.19  (0.19) | | -0.02  (0.14) | | | -0.00  (0.12) | | | -0.02  (0.18) | | | -0.02  (0.20) | | | -0.49**  (0.18) | | | -0.16  (0.20) | | | |  |
| Non-centrist party x support | -0.09  (0.29) | | -0.04  (0.31) | | 0.03  (0.30) | | 0.00  (0.19) | | | 0.27  (0.18) | | | 0.42  (0.24) | | | -0.20  (0.29) | | | 0.54†  (0.26) | | | 0.20  (0.25) | | | |  |
| *R*^2^ | .106 | .061 | | .228 | | .123 | | | .096 | | | .145 | | | .283 | | | .340 | | | .202 | | | |  |  |

*Note*. *N* = 2,007. Cells show unstandardized regression coefficients with standard errors in parentheses. PEAA = perceived expectations for active aging. PH = physical health domain. MH = mental health domain. SE = social engagement domain. SES = subjective socioeconomic status. Income difference was standardized.

^a^ Reference group: occupations less disrupted by the pandemic.

^b^ Reference group: centrist-party preference.

† *p* < .10 * *p* < .05. ** *p* < .01. *** *p* < .001. (for planned significance tests)

† *p* < .05. * *p* < .01. ** *p* < .001. (for post-hoc significance tests, including those for control variables)

**Article title**: Did aging-related beliefs and behaviors change during the COVID-19 pandemic?

**Journal name**: European Journal of Ageing

**Author names and affiliation**: Sonja Radoš, M.Sc. (University of Vechta), Maria K. Pavlova, Ph.D. (University of Vechta), Klaus Rothermund, Ph.D. (Friedrich Schiller University of Jena), and Rainer K. Silbereisen, Ph.D. (Friedrich Schiller University of Jena)

**Corresponding author**: Sonja Radoš, [sonja.rados@uni-vechta.de](mailto:sonja.rados@uni-vechta.de)
